# Distal Arthrogryposis type 5 in an Italian family due to an autosomal dominant gain-of-function mutation of the *PIEZO2* gene

**DOI:** 10.1186/s13052-022-01329-z

**Published:** 2022-07-29

**Authors:** Gregorio Serra, Vincenzo Antona, Chiara Cannata, Mario Giuffrè, Ettore Piro, Ingrid Anne Mandy Schierz, Giovanni Corsello

**Affiliations:** Department of Health Promotion, Mother and Child Care, Internal Medicine and Medical Specialties “G. D’Alessandro”, University of Palermo, Palermo, Italy

**Keywords:** Arthrogryposis multiplex congenita, DA5, Ophthalmoplegia, *PIEZO2* gene, Gain-of-function mutation, NGS, Case report

## Abstract

**Background:**

Arthrogryposis multiplex congenita (AMC) is a group of clinically and etiologically heterogeneous conditions, characterized by prenatal onset contractures affecting two or more joints. Its incidence is about 1 in 3000 live births. AMC may be distinguished into amyoplasia, distal and syndromic arthrogryposis. Distal arthrogryposis (DA) predominantly affects hands and feet. It is currently divided into more than ten subtypes (DA1, DA2A/B, DA3–10), based on clinical manifestations, gene mutations and inheritance pattern. Among them, only a few patients with DA5 have been reported. It is associated to a gain-of-function pathogenic variant of the *PIEZO2* gene, encoding for an ion-channel necessary to convert mechanical stimulus to biological signals and crucial for the development of joints, neuromuscular and respiratory systems. Main clinical features include multiple distal contractures, short stature, ptosis, ophthalmoplegia and, in some cases, restrictive lung disease.

**Case presentation:**

Hereby, we report on a four-generation Italian family with DA5. Our first proband was a newborn with prenatal suspicion of AMC. At birth, clinical findings were compatible with a DA diagnosis. Family history was positive for the mother with *short stature,* ophthalmoplegia, short neck, and contractures of the joints of distal extremities, and for three other relatives on the maternal side, including grandfather and great-grandmother, who all shared similar findings. Thus, we performed a next generation sequencing analysis (NGS) of the genes associated to AMC and of those involved in DA. The gain-of-function heterozygous mutation c.8181_8183delAGA (p.Glu2727del) of *PIEZO2* was identified in the proband, and the same mutation was also found in the mother, confirming the autosomal dominant inheritance of the condition.

**Conclusions:**

Our patients contribute to the current DA5 genomic database, and to a better characterization of the disease. Clinicians may have suspicion of a DA diagnosis based on suggestive (also prenatal) clinical findings, which must be then confirmed by NGS analysis. Since natural history varies widely among different DA disorders, detection of the underlying causal variant is essential for the identification of the exact subtype, and to its adequate management, which must rely on a multidisciplinary and individualized approach.

## Background

Arthrogryposis Multiplex Congenita (AMC) is a group of clinically and etiologically heterogeneous conditions characterized by prenatal onset contractures affecting two or more joints. The incidence is estimated at about 1 in 3000 live births, with female to male ratio 1:1 [1]. The pathogenic mechanism underlying arthrogryposis is the reduction of fetal movements, leading to an atypical increase of connective tissue around the joints (collagenosis) during development. This, in turn, further limits the joint movement and increases the contractures [2]. AMC has been described as a clinical feature in more than 400 specific disorders, and over 400 genes are currently associated to arthrogryposis [1, 2]. AMC may be classified into amyoplasia, distal (DA) and syndromic arthrogryposis [3]. DA predominantly affects hands and feet, and more than ten subtypes (DA1, DA2A and B, DA3–10) have been reported, based on clinical manifestations (including extra-articular findings), as well as gene pathogenic variants and inheritance pattern [1]. Distal arthrogryposis type 5 (DA5, MIM#108145) shows autosomal dominant inheritance, and its clinical features include multiple distal contractures, short stature, triangular face, ocular manifestations including deep-set eyes, ptosis and ophthalmoplegia, a textural peculiarity of the muscles to palpation described as “woody”, and in some cases restrictive lung disease with pulmonary hypertension [2]. It is associated to a gain-of-function heterozygous variant of the *PIEZO2* (piezo type mechanosensitive ion channel type 2) gene, encoding for an ion-channel protein necessary to convert mechanical stimulus to biological signals and crucial for the development of joints, and neuromuscular and respiratory systems [4]. Only a few cases of DA5 have been described to date, although such condition is sometimes mistaken with the allelic phenotypes of *PIEZO2*, namely Gordon (GS) and Marden-Walker (MWS) syndromes, and/or with other DAs subtypes [5]. Hereby, we report on an Italian family affected with DA5, in which target next generation sequencing (NGS) analysis revealed the pathogenic gain-of-function heterozygous variant c.8181_8183delAGA (p.Glu2727del) of the *PIEZO2* gene.

## Case presentation

**Fig. 1:**
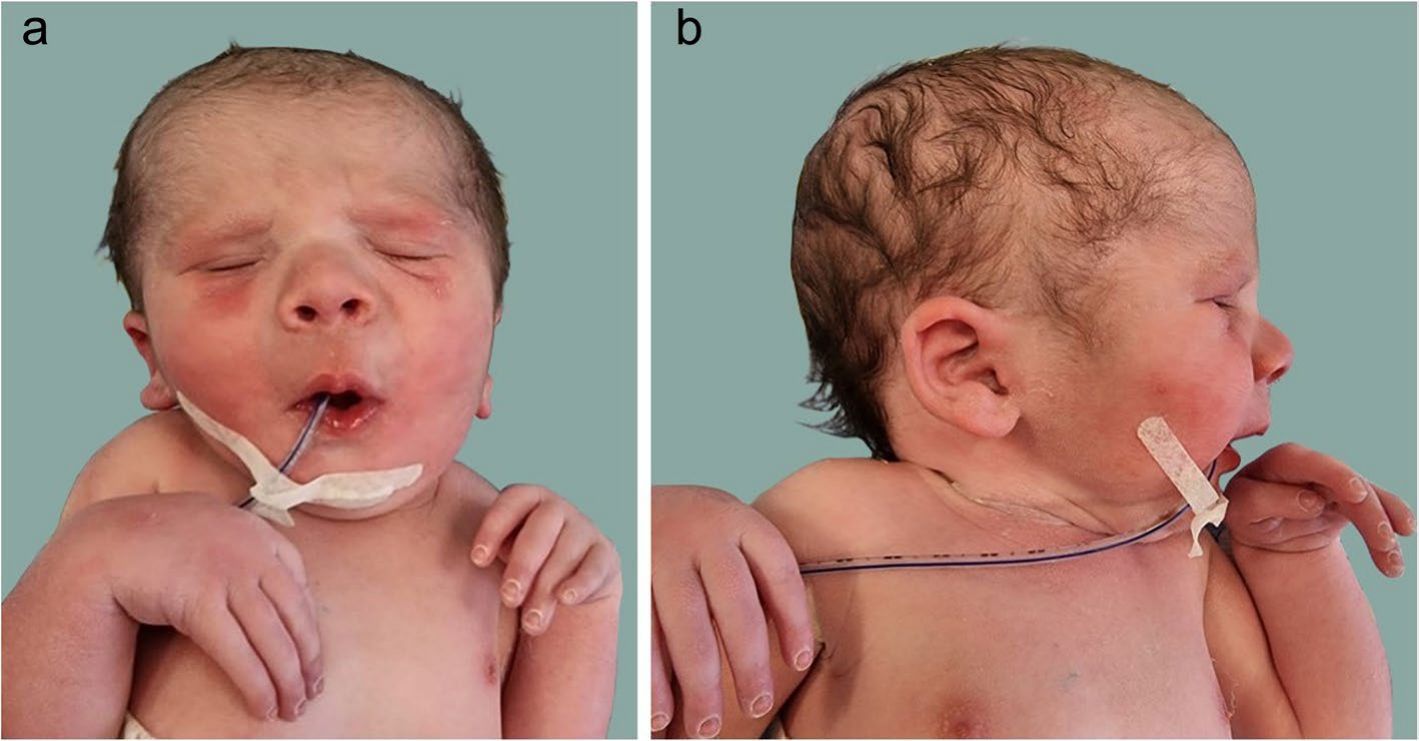
**a** Patient’s front view. High forehead, low anterior hairline, deep-set eyes, wide and depressed nasal bridge, bulbous nose, anteverted nares, long and thick philtrum, increased nasogenian folds and half-opened mouth with “whistling” appearance. **b**. Lateral view. Right posteriorly rotated ear with thick helix, and microretrognathia

**Fig. 2:**
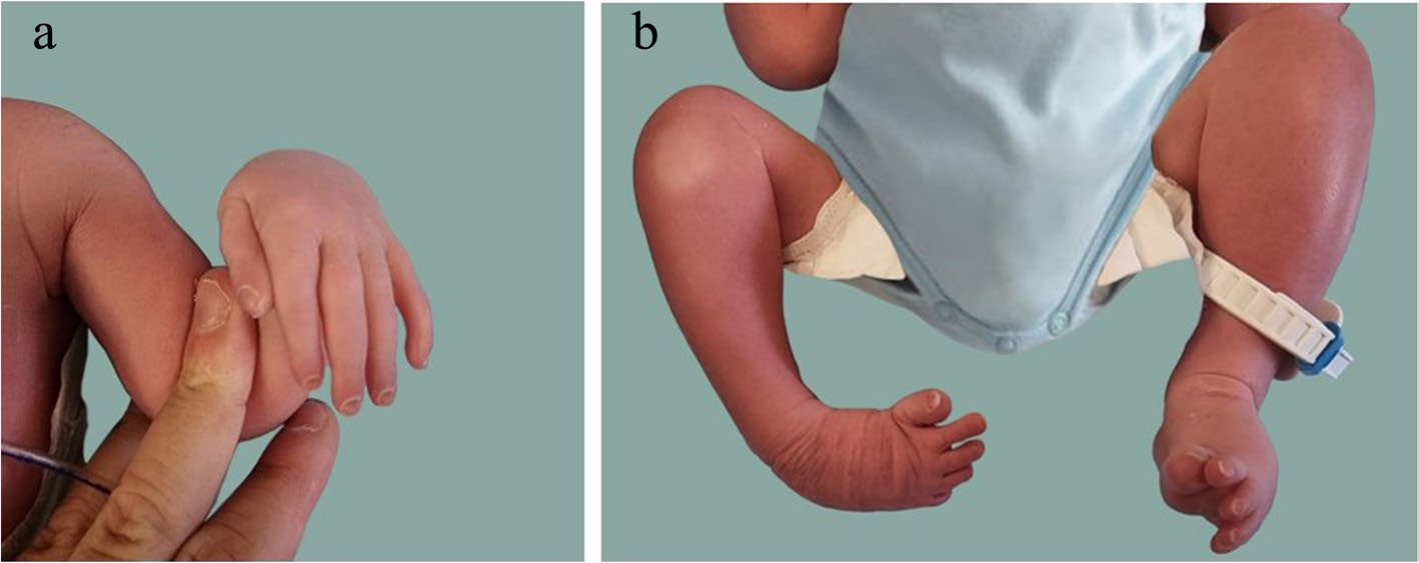
**a** Ulnar deviation of the hand, arachnodactyly, proximal set and short first, and fifth finger (showing also clinodactyly). **b** Talipes *equinus-varus-adductus-supinatus*, overlapping toes, with short, and proximal set of the first (also straight and broad) and fifth ones

A male newborn, first child of Italian nonconsanguineous parents, was born at 38 + 1 weeks of gestation by caesarean section due to preeclampsia. Pregnancy was complicated by hypertension treated with methyldopa. Second trimester prenatal ultrasound (US) revealed oligohydramnios, flexed wrists, and bilateral clubfeet, raising the diagnostic suspicion of AMC. Apgar scores were 8, 8 and 9 at 1, 5 and 10 minutes respectively. At birth, anthropometric measurements were as follows: weight 2460 g (5th centile, − 1.65 standard deviation, SD), length 47 cm (12th centile, − 1.17 SD) and occipitofrontal circumference (OFC) 36 cm (95th centile, + 1.65 SD). Soon after birth, he was transferred to the neonatal intensive care unit due to mild respiratory distress, that required non-invasive ventilatory support by continuous positive airway pressure. At admission, physical examination showed high forehead, low anterior hairline, deep-set eyes, wide and depressed nasal bridge, bulbous nose, anteverted nares, long and thick philtrum, increased nasogenian folds and half-opened mouth with “whistling” appearance. The right posteriorly rotated ear with bilateral thick helix, and microretrognathia completed his craniofacial profile (Fig. 1a, b). *Pectus excavatum*, and increased tone (“woody”) of the muscles of the abdominal wall were also observed. Anomalies of the extremities included ulnar deviation of the hands, bilateral arachnodactyly, proximal set and short first and fifth fingers, with clinodactyly of the latters, in addition to talipes *equinus-varus-adductus-supinatus*, with overlapping toes, short and proximal set of the first (also straight and broad) and fifth toes (Fig. 2a, b). Neurological findings were a mild generalized hypotonia, poor reactivity, crying and suction, as well as decreased osteotendinous and archaic reflexes. Most of these phenotypic features were observed in the mother, who had short stature (height 150 cm), ophthalmoplegia, short neck, along with contractures of the joints of distal extremities. Furthermore, family history disclosed three further relatives (grandfather, aunt, and great-grandmother), on the maternal side, sharing overlapping clinical features.

The clinical course was characterized by the need of non-invasive ventilatory support during the first week of life. Due to lack of sucking/swallowing coordination, nasogastric tube feeding was initially required. Laboratory analyses including complete blood count, serum electrolytes, liver, kidney, and thyroid function tests showed normal results. Ophthalmologic examination revealed a bilateral decreased accommodation reflex, secondary to ophthalmoplegia. Except for mild enlargement of the left ventricle, major structural brain anomalies were ruled out on head US. Moreover, abdominal US documented no abnormalities, and the echocardiographic evaluation, revealed an isolated patent *foramen ovale*. Conversely, skeletal X-Ray confirmed the clinically observed abnormalities of the extremities, consisting of ulnar deviation of the hands, talipes *equinus-varus-adductus-supinatus*, in addition to proximal set and short first and fifth fingers and toes. No bone anomalies were identified in the proximal segments of the extremities, chest, spine and hips. Then, having considered the family history along with the clinical, laboratory and image findings, a targeted next generation sequencing analysis (NGS) of the genes associated to AMC and of those involved in distal arthrogryposis and digital synostosis (Table 1) was performed. The gain-of-function heterozygous pathogenic variant c.8181_8183delAGA (p.Glu2727del) (Ref Seq NM_022068.3, based on genome build GRCh37/hg19) of the *PIEZO2* gene was identified in the proband, and the same mutation was also found in his mother. Genetic investigations of the other family members were not carried out due to restrictions related to the COVID-19 pandemic emergency occurring at the time of the hospital stay of our patient.

**Table 1:** Genes included and quality of target NGS analysis

Name HGNC	Full name	OMIM	Coding sequence length (bases number)	>5x	Coverage% > 10x	>20x	Depth of medium	coverage (x) maximum
**Arthrogryposis multiplex congenita**								
*ADCY6*	Adenylate cyclase 6	600,294	3507	100.00	100.00	100.00	380.13	832
*ASCC1*	Activating signal cointegrator 1 complex subunit 1	614,215	1203	100.00	100.00	99.17	172.73	423
*CNTN1*	Contactin 1	600,016	3024	100.00	100.00	100.00	196.61	458
*CNTNAP1*	Contactin associated protein 1	602,346	4155	100.00	100.00	100.00	358.82	923
*DOK7*	Docking protein 7	610,285	1515	100.00	99.74	97.43	331.79	736
*ERGIC1*	Endoplasmic reticulum-golgi intermediate compartment 1	617,946	873	100.00	100.00	100.00	444.82	747
*FKBP10*	FKBP prolyl isomerase 10	607,063	1749	100.00	100.00	100.00	359.79	769
*GLE1*	GLE1 RNA export mediator	603,371	2097	100.00	100.00	100.00	194.62	387
*KIF14*	Kinesin family member 14	611,279	4947	100.00	100.00	100.00	167.48	323
*LGI14*	Leucine rich repeat LGI family member 4	608,303	1614	100.00	100.00	100.00	222.95	429
*MUSK*	Muscle associated receptor tyrosine kinase	601,296	2610	100.00	100.00	100.00	222.38	508
*MYBPC1*	Myosin binding protein C, slow type	160,794	3522	100.00	100.00	100.00	183.71	581
*MYOD1*	Myogenic differentiation 1	159,970	963	100.00	100.00	100.00	623.24	1438
*NUP88*	Nucleoporin 88	602,552	2226	100.00	100.00	100.00	206.06	482
*PIEZO2*	Piezo type mechanosensitive ion channel component 2	613,629	8259	100.00	100.00	99.94	191.65	677
*RAPSN*	Receptor associated protein of the synapse	601,592	1239	100.00	100.00	100.00	419.22	935
*SCARF2*	Scavenger receptor class F member 2	613,619	2598	100.00	100.00	100.00	209.97	618
*SYNE1*	Spectrin repeat containing nuclear envelope protein 1	608,441	26,394	100.00	100.00	100.00	193.42	680
*TRIP4*	Thyroid hormone receptor interactor 4	604,501	1746	100.00	100.00	100.00	140.63	238
*UBA1*	Ubiquitin like modifier activating enzyme 1	314,370	3177	100.00	100.00	100.00	215.59	532
*VIPAS39*	VPS33B late endosome and lysosome associated	608,552	1854	100.00	100.00	100.00	218.34	597
*ZC4H2*	Zinc finger C4H2-type containing	300,897	675	100.00	100.00	100.00	103.71	158
**Distal arthrogryposis**								
*CHST14*	Carbohydrate sulfotransferase 14	608,429	1131	100.00	100.00	100.00	260.38	467
*DSE*	Dermatan sulfate epimerase	605,942	2877	100.00	100.00	100.00	206.13	547
*ECEL1*	Endothelin converting enzyme like 1	605,896	2328	100.00	100.00	98.80	248.47	929
*FBN2*	Fibrillin 2	612,570	8739	100.00	100.00	100.00	225.84	600
*MYBPC1*	Myosin binding protein C, slow type	160,794	3522	100.00	100.00	100.00	183.71	581
*MYH3*	Myosin heavy chain 3	160,720	5823	100.00	100.00	100.00	211.55	551
*MYH8*	Myosin heavy chain 8	160,741	5814	100.00	100.00	99.47	191.77	746
*NALCN*	Sodium leak channel, non-selective	611,549	5217	100.00	100.00	100.00	177.57	395
*PIEZO2*	Piezo type mechanosensitive ion channel component 2	613,629	8259	100.00	100.00	99.94	191.65	677
*SLC35A3*	Solute carrier family 35 member A3	605,632	1104	100.00	100.00	100.00	156.35	264
*TNNI2*	Troponin I2, fast skeletal type	191,043	549	100.00	100.00	100.00	355.52	890
*TNNT1*	Troponin T1, slow skeletal type	191,041	837	100.00	100.00	100.00	196.36	370
**Other genes**								
*ACTA1*	Actin alpha 1, skeletal muscle	102,610	1134	100.00	100.00	100.00	250.29	714
*AGRN*	Agrin	103,320	6138	100.00	100.00	99.54	351.00	897
*BIN1*	Bridging integrator 1	601,248	1782	100.00	100.00	100.00	283.67	580
*CASK*	Calcium/calmodulin dependent serine protein kinase	300,172	2766	100.00	100.00	100.00	110.05	307
*CFL2*	Cofilin 2	601,443	501	100.00	100.00	100.00	142.68	262
*CHAT*	Choline O-acetyltransferase	118,490	2247	100.00	100.00	98.00	234.82	481
*CHRNA1*	Cholinergic receptor nicotinic alpha 1 subunit	100,690	1374	100.00	100.00	100.00	219.81	422
*CHRNB1*	Cholinergic receptor nicotinic beta 1 subunit	100,710	1506	100.00	100.00	100.00	257.88	591
*CHRND*	Cholinergic receptor nicotinic delta subunit	100,720	1554	100.00	100.00	100.00	326.22	666
*CHRNE*	Cholinergic receptor nicotinic epsilon subunit	100,725	1482	100.00	100.00	100.00	311.97	705
*CHRNG*	Cholinergic receptor nicotinic gamma subunit	100,730	1554	100.00	100.00	100.00	309.39	644
*COL6A2*	Collagen type VI alpha 2 chain	120,240	3060	100.00	100.00	100.00	380.62	701
*COLQ*	Collagen like tail subunit of asymmetric acetylcholinesterase	603,033	1368	100.00	100.00	100.00	193.63	454
*DHCR24*	24-dehydrocholesterol reductase	606,418	1551	100.00	100.00	100.00	305.51	710
*DPAGT1*	Dolichyl-phosphate N-acetylglucosaminephosphotransferase 1	191,350	1227	100.00	100.00	100.00	209.58	349
*EGR2*	Early growth response 2	129,010	1431	100.00	100.00	100.00	313.71	523
*ERCC5*	ERCC excision repair 5, endonuclease	133,530	3561	100.00	100.00	100.00	171.74	386
*ERCC6*	ERCC excision repair 6, chromatin remodeling factor	609,413	4482	100.00	100.00	100.00	229.78	501
*EXOSC3*	Exosome component 3	606,489	828	100.00	100.00	99.88	292.51	690
*FHL1*	Four and a half LIM domains 1	300,163	972	100.00	100.00	98.66	96.30	207
*FKTN*	Fukutin	607,440	1386	100.00	100.00	100.00	198.58	376
*GBA*	Glucosylceramidase beta	606,463	1611	100.00	100.00	100.00	602.97	1174
*GBE1*	1,4-alpha-glucan branching enzyme 1	607,839	2109	100.00	100.00	100.00	204.50	501
*GFPT1*	Glutamine-fructose-6-phosphate transaminase 1	138,292	2100	100.00	100.00	100.00	162.87	429
*GLDN*	Gliomedin	608,603	1656	100.00	100.00	100.00	157.95	407
*KAT6B*	Lysine acetyltransferase 6B	605,880	6222	100.00	100.00	100.00	276.88	1144
*KLHL40*	Kelch like family member 40	615,340	1866	100.00	100.00	100.00	307.58	611
*MPZ*	Myelin protein zero	159,440	747	100.00	100.00	100.00	293.78	823
*MTM1*	Myotubularin 1	300,415	1812	100.00	100.00	100.00	111.02	238
*MYH2*	Myosin heavy chain 2	160,740	5826	100.00	100.00	100.00	200.07	480
*NEB*	Nebulin	161,650	19,974	100.00	100.00	100.00	193.18	564
*PLOD2*	Procollagen-lysine,2-oxoglutarate 5-dioxygenase 2	601,865	2277	100.00	100.00	100.00	166.51	435
*PMM2*	Phosphomannomutase 2	601,785	741	100.00	100.00	100.00	202.86	369
*RARS2*	Arginyl-tRNA synthetase 2, mitochondrial	611,524	1737	100.00	100.00	100.00	164.02	377
*SCO2*	SCO cytochrome c oxidase assembly protein 2	604,272	801	100.00	100.00	100.00	325.72	627
*TGFB3*	Transforming growth factor beta 3	190,230	1239	100.00	100.00	100.00	280.94	449
*TK2*	Thymidine kinase 2	188,250	798	100.00	100.00	99.25	212.26	474
*TNNT3*	Troponin T3, fast skeletal type	600,692	771	100.00	100.00	100.00	286.03	564
*TPM3*	Tropomyosin 3	191,030	858	100.00	100.00	100.00	181.98	368
*TRPV4*	Transient receptor potential cation channel subfamily V member 4	605,427	2616	100.00	100.00	100.00	308.90	554
*TSEN2*	tRNA splicing endonuclease subunit 2	608,753	1398	100.00	100.00	100.00	223.97	579
*TSEN54*	tRNA splicing endonuclease subunit 54	608,755	1581	100.00	99.11	97.28	273.41	462
*VRK1*	VRK serine/threonine kinase 1	602,168	1191	100.00	100.00	100.00	177.52	298
*ZBTB42*	Zinc finger and BTB domain containing 42	613,915	1269	100.00	100.00	100.00	372.32	655
**Digital synostosis**								
*BHLHA9*	Basic helix-loop-helix family member a9	615,416	708	100.00	89.27	81.50	135.39	384
*BMPR1B*	Bone morphogenetic protein receptor type 1B	603,248	1599	100.00	100.00	100.00	210.72	332
*CHSY1*	Chondroitin sulfate synthase 1	608,183	2409	100.00	99.63	98.13	215.28	398
*FGF9*	Fibroblast growth factor 9	600,921	627	100.00	100.00	100.00	187.26	287
*GDF5*	Growth differentiation factor 5	601,146	1506	100.00	100.00	100.00	364.68	699
*GDF6*	Growth differentiation factor 6	601,147	1368	100.00	100.00	100.00	262.93	704
*IHH*	Indian hedgehog signaling molecule	600,726	1236	100.00	100.00	100.00	303.92	420
*NOG*	Noggin	602,991	699	100.00	100.00	100.00	416.59	809
*PCNT*	Pericentrin	605,925	10,011	100.00	100.00	99.91	207.88	569
*PTDSS1*	Phosphatidylserine synthase 1	612,792	1422	100.00	100.00	100.00	197.51	399vements restriction

In the following months, the proband showed mild generalized hypotonia and developmental delay. However, he overcame his initial feeding difficulties, reaching adequate and exclusive bottle feeding with standard infant formula, at around 3 weeks of life. He was discharged from the Hospital at about 1 month of age, in good general condition but with poor weight gain and growth, and included in a multidisciplinary follow-up. Initial hearing screening, through transient evoked otoacoustic emissions (TEOAEs), showed abnormal results. To ascertain and characterize the hearing loss, an audiological assessment was started. It included brain auditory evoked response (BAER) evaluation at 3 months of age, which detected bilateral response threshold at 30 dB (decibel) HL (hearing level) according to mild hearing loss, that did not require any treatment. He underwent further ophthalmological assessments, which confirmed the previous findings compatible with ophthalmoplegia. He also performed hip US, which ruled out congenital dysplasia. Finally, an orthopedic evaluation was carried out, which counseled and prescribed the conservative Ponseti method for the management of bilateral clubfoot, consisting in manipulation, serial casting, and Achilles tendon tenotomy followed by foot abduction bracing. Indeed, he underwent reduction of the right foot deformity with plaster casting, and a percutaneous Achilles tenotomy is at present planned.

The proband is now 4 months and 6 days old, and shows a poor growth: weight Kg 5.020 (<3rd centile, − 3 SD), length 58 cm (<3rd centile, − 3.01 SD), and head circumference 40.5 cm (14th centile, − 1.09 SD) (according to World Health Organization growth standards for neonatal and infant close monitoring) [6]. The child is presently placed in a rehabilitation program, including physiokynesiotherapy as well as occupational and manipulation treatment of the upper limbs, to improve the hands contractures. He has increased axial, upper and lower limbs and abdominal muscles’ tone, and delayed motor development. Clinical examination and multiorgan US evaluations showed no further anomalies.

## Discussion and conclusions

DA was first classified by Hall, Reed, and Greene, as a heterogeneous group of disorders with congenital joint contractures, predominantly affecting hands and feet. Although originally described as autosomal dominant (AD) trait, it is well known that DA may also show autosomal recessive (AR) pattern of transmission [1, 2].

DA is presently classified into more than ten subtypes (DA1, DA2A and B, and DA3–10), depending on the pattern of contractures combined with extraarticular features [7]. Distal arthrogryposis type 5 (DA5), originally classified as type 2B, is characterized by short stature, characteristic *facies* with ocular manifestations, and AD trait [8, 9]. Nevertheless, other features have been added to the phenotype, including ophthalmoplegia, pulmonary dysfunction, and a textural peculiarity of the muscles to palpation, described as “woody”. Its genotype was first identified by Coste et al. [7], through NGS, in three patients with the aforementioned clinical features and a heterozygous variant of *PIEZO2*. Such gene encodes for a large transmembrane protein (named from the Greek term πιεση, meaning pressure), belonging to components of mechanically (MA) or stretch-activated ion channels, found in many cells and tissues/organs (somatosensory neurons, dorsal root ganglions, inner ear hair, muscle and endothelial cells, osteoblasts, cartilage, urinary bladder, lungs, kidneys, and gastrointestinal tract) [4]. Its action allows the phenomenon of mechanic transduction, which is the translation of mechanical force into biochemical signals. Therefore, it plays crucial roles in different processes, including perception and proprioception, pain and hearing, and further potential ones are assumed for the development of the skeletal, neuromuscular and respiratory systems during embryogenesis [10]. Indeed, the identification of *PIEZO2* pathogenic variants in DA5, as in the present family, has provided further insights into the potential pathogenic mechanisms of the disease [11]. Specifically, its clinical picture may be related to gain-of-function pathogenic variants leading to hyperactive PIEZO2 signaling and increased channel activity, which may decrease joint extension, lung or thorax expansion, and ocular movement (muscular fibrosis leading to contractures may be the cause of ophthalmoparesis) [12, 13]. It is uncertain whether the respiratory complications are age dependent [14]. The current absence of chest and lung involvement in the mother of our newborn may not rule out its possible appearance over time.

To date, *PIEZO2* missense, and frameshift (as the one here described, rsID 587,777,077, Ensembl transcript ENST00000503781.7, and reported in literature by some Authors [5, 7, 10]) pathogenic variants, account for the vast majority of variants. They have highly pleomorphic effects and different pathophysiological consequences [15, 16]. The clinical manifestations of *PIEZO2*-associated diseases display a great variation, as well [10]. Indeed, gain-of-function mutations of *PIEZO2* have been also linked with DA3 (also known as Gordon Syndrome, GS, MIM#114300), Marden-Walker Syndrome (MWS, MIM#248700) and other related diseases [12, 17]. GS is commonly mistaken with DA5, but it may be distinguished by the presence of cleft palate and bifid uvula, whereas ophthalmological, muscle, and respiratory problems are primarily observed in DA5 [15]. Other less frequent signs and symptoms seen in DA5 patients are *pectus excavatum* (33%, observed also in our patient), *trismus* (26%), metacarpal and metatarsal synostosis (25%), toe syndactyly (18%), neck webbing (8%, found in the mother of our newborn), and sensorineural hearing loss (6%, and also present in the proband) [15]. Differential diagnosis of DA5 also includes Aase-Smith Syndrome (MIM#147800), and Marden-Walker Syndrome (characterized by joint contractures, cleft palate, blepharophimosis, “immobile” *facies*, diminished muscular bulk, developmental delay and hindbrain malformations) [15].

Hereby, we report on a four-generation family with clinical pictures compatible with DA5, in which two members (the newborn proband and his mother) were found to have the same gain-of-function heterozygous pathogenic variant of *PIEZO2*. The present study contributes to the current genomic databases, and to a better characterization of the disease. Moreover, it highlights the age-dependent phenotypic variability, which may also be observed among family members.

Clinicians may suspect DA based on suggestive (also prenatal) clinical findings, which must be then confirmed by NGS analysis [18–22]. Since natural history varies widely among different DA disorders, identification of the underlying causal variant is essential. The existing classification of DAs is a helpful tool for the differential diagnosis. Indeed, the prompt recognition of signs and symptoms of DA in our patient, in addition to NGS analysis, has led to early identification of the exact subtype (DA5), and then to proper management.

Comorbidities and/or potential complications related to growth, feeding, development and behavior, musculoskeletal system, ophthalmological abnormalities, respiratory difficulties, and hearing defects should be prevented and/or reduced according to a multidisciplinary and individualized approach [23–26]. Enrollment in physical and occupational therapy may improve the fine motor skills in these subjects. Periodic ophthalmological examinations are recommended to rule out keratoconus, refraction problems or abnormalities of the retina, which may require correction, while hearing screening is able to early detect sensorineural hearing loss (as in our proband). Moreover, pulmonary function testing and echocardiography should be performed for the early diagnosis of restrictive pulmonary disease [15].

Further understanding of the physiological implications of gain-of-function mutations of *PIEZO2* is required to find the most effective management and treatment for each patient, and ultimately to improve the quality of life among patients with DA5 and *PIEZO2*-related phenotypes.

## Data Availability

The datasets used and analyzed during the current study are available from the corresponding author on reasonable request.

## Abbreviations

AMC: Arthrogryposis Multiplex Congenita
BAER: Brain Auditory Evoked Response
DA: Distal Arthrogryposis
GS: Gordon Syndrome
MA: Mechanically Activated
MWS: Marden-Walker Syndrome
NGS: Next Generation Sequencing
OFC: Occipitofrontal Circumference
*PIEZO2*: Piezo type mechanosensitive ion channel type 2
TEOAE: Transient-Evoked OtoAcoustic Emissions
US: UltraSonography
